# Supplementary material for: Soft Tissue Sarcoma Mimicking Melanoma: A Systematic Review
Source: Cancers (Basel). 2023 Jul 12;15(14):3584. doi: 10.3390/cancers15143584 (PMC10377019; doi:10.3390/cancers15143584)
Supplement: Supplementary file 1 [file cancers-15-03584-s001.zip › cancers-2488449-supplementary.pdf]

Supplementary Table S1. List of excluded records after reading the full-text.

Record	Reason for exclusion
Ferreira I, Arends MJ, van der Weyden L, Adams DJ, Brenn T. Primary de-differentiated, trans-differentiated and undifferentiated melanomas: overview of the clinicopathological, immunohistochemical and molecular spectrum. Histopathology. 2022;80(1):135-149.	Different design
Hantschke M, Mentzel T, Rütten A, Palmedo G, Calonje E, Lazar AJ, Kutzner H. Cutaneous clear cell sarcoma: a clinicopathologic, immunohistochemical, and molecular analysis of 12 cases emphasizing its distinction from dermal melanoma. Am J Surg Pathol. 2010;34(2):216-22.	Different design
Allanson BM, Weber MA, Jackett LA, Chan C, Lau L, Ziegler DS, Warby M, Mayoh C, Cowley MJ, Tucker KM, Long GV, Maher A, Anazodo A, Scolyer RA. Oral malignant gastrointestinal neuroectodermal tumour with junctional component mimicking mucosal melanoma. Pathology. 2018;50(6):648-653.	Different design
Epstein AL, Martin AO, Kempson R. Use of a newly established human cell line (SU-CCS-1) to demonstrate the relationship of clear cell sarcoma to malignant melanoma. Cancer Res. 1984;44(3):1265-74.	Different design
Ko CJ, Bolognia JL, Glusac EJ. "Clark/dysplastic" nevi with florid fibroplasia associated with pseudomelanomatous features. J Am Acad Dermatol. 2011;64(2):346-51.	Different topic
Kim TJ, Kim HJ, Cho MK, Lee SY, Lee JS, Whang KU. A case of common blue nevus with malignant melanoma-like satellite lesions.Korean Journal of Dermatology. 2022;40(11):1426-28.	Different topic
Sugiyama M, Arakawa A, Kumamoto T, Aoki Y, Sonoda T, Ishimaru S, Shirakawa N, Ohyama R, Keino D, Kinoshita A, Ogawa C. A case of malignant melanoma-like spindle sarcoma treated with nivolumab and trabectedin. Pediatric Blood and Cancer. Conference: 59th Annual Meeting of the Japanese Society of Pediatric Hematology and Oncology. Matsuyama City Japan. 2017;64(Supplement 4):S41.	Different topic
Morariu SH, Suciu M, Vartolomei MD, Badea MA, Cotoi OS. Aneurysmal dermatofibroma mimicking both clinical and dermoscopic malignant melanoma and Kaposi's sarcoma. Rom J Morphol Embryol. 2014;55(3 Suppl):1221-4.	Different topic
Liedtke KR, Waldburger N, Glitsch AS, Schreiber A. Castleman's disease mimicked melanoma metastasis in the mesentery - A case report. Int J Surg Case Rep. 2020;67:110-113.	Different topic
Furuya M, Shimizu M, Nishihara H, Ito T, Sakuragi N, Ishikura H, Yoshiki T. Clear cell variant of malignant melanoma of the uterine cervix: a case report and review of the literature. Gynecol Oncol. 2001;80(3):409-12.	Different topic
Zappettini E, Castilla S, Valente B, Cucci A, Fernández S. Clear cell sarcoma mimicking a breast tumor in an elderly man: a rare case report and a literature review. WCRJ 2022; 9: e2250	Different topic
Yousef S, Joy C, Velaiutham S, Maclean FM, Harraway J, Gill AJ, Vargas AC. Dedifferentiated melanoma with MDM2 gene amplification mimicking dedifferentiated liposarcoma. Pathology. 2022;54(3):371-374.	Different topic
Tripathy S, Mishra L, Baisakh M, Mohapatra N, Das S. Giant eccrine spiradenoma mimicking a malignant tumor. Indian J Dermatol Venereol Leprol. 2015;81(1):79-80.	Different topic
Laureano A, Fernandes C, Cardoso J. Hemosiderotic dermatofibroma: clinical and dermoscopic presentation mimicking melanoma. J Dermatol Case Rep. 2015;9(2):39-41.	Different topic
Lancer HA, Bronstein BR, Sober AJ. Multiple cutaneous melanoma metastases of an extremity resembling Kaposi's sarcoma: use of regional perfusion. J Dermatol Surg Oncol. 1984;10(3):196-9.	Different topic
Vaccari S, La Placa M, Barisani A, Lacava R, Misciali C, Tosti G, Gaspari V. Penile Exogenous Pigmentation Mimicking Melanoma. Dermatol Pract Concept. 2022;12(1):e2022014.	Different topic
Zilakos NP, Tsonis PA. A spontaneous melanoma-like tumor in the adult newt Triturus cristatus. Tumour Biol. 1991;12(2):120-4.	Different participants (animal study)
Wechsler J, Guillaume JC, Baspeyras M, Revuz J. Sarcome épithélioïde simulant un mélanome du pied [Epithelioid sarcoma mimicking melanoma of the foot]. Ann Dermatol Venereol. 1990;117(11):871-2.	Full-text unavailable or study not completed
Russo A. Unifocal skin location of Kaposi's sarcoma, mimicking melanoma: the role of excisional biopsy for the timely identification of an unusual pathology with potential fatal prognosis. G Chir. 2001;22(8-9):285-9.	Full-text unavailable or study not completed
